# Disentangling the Effects of Precipitation Amount and Frequency on the Performance of 14 Grassland Species

**DOI:** 10.1371/journal.pone.0162310

**Published:** 2016-09-13

**Authors:** Teresa J. Didiano, Marc T. J. Johnson, Tim P. Duval

**Affiliations:** 1 Department of Geography, University of Toronto Mississauga, Mississauga, Ontario, Canada; 2 Department of Biology, University of Toronto Mississauga, Mississauga, Ontario, Canada; Instituto Agricultura Sostenible, SPAIN

## Abstract

Climate change is causing shifts in the amount and frequency of precipitation in many regions, which is expected to have implications for plant performance. Most research has examined the impacts of the amount of precipitation on plants rather than the effects of both the amount and frequency of precipitation. To understand how climate-driven changes in precipitation can affect grassland plants, we asked: (i) How does the amount and frequency of precipitation affect plant performance? (ii) Do plant functional groups vary in their response to variable precipitation? To answer these questions we grew 14 monocot and eudicot grassland species and conducted a factorial manipulation of the amount (70 vs 90mm/month) and frequency (every 3, 15, or 30 days) of precipitation under rainout shelters. Our results show that both the amount and frequency of precipitation impact plant performance, with larger effects on eudicots than monocots. Above- and below-ground biomass were affected by the amount of precipitation and/or the interaction between the amount and frequency of precipitation. Above-ground biomass increased by 21–30% when the amount of precipitation was increased. When event frequency was decreased from 3 to 15 or 30 days, below-ground biomass generally decreased by 18–34% in the 70 mm treatment, but increased by 33–40% in the 90 mm treatment. Changes in stomatal conductance were largely driven by changes in event frequency. Our results show that it is important to consider changes in both the amount and frequency of precipitation when predicting how plant communities will respond to variable precipitation.

## Introduction

Within the next century, climate change will cause alterations in annual and seasonal precipitation, as well as increased precipitation variability [1–3]. In some regions throughout the world, summer precipitation regimes will be characterized by large, infrequent precipitation events with longer intervening dry periods [1, 4, 5]. Changing the size and timing of precipitation events will introduce novel hydrological conditions that could influence plant community dynamics [6–8], and studying these processes remains a key challenge to understanding the ecosystem-level consequences of climate change [9–11]. Here, we address this challenge by examining the individual and interactive effects that differences in the amount and frequency of precipitation have on plant performance of grassland monocot and eudicot species.

Plant performance can be altered by changes in water availability [12, 13]. Precipitation regimes that are characterized by large, infrequent events separated by dry periods can drive transitions in grassland systems from states of low water stress (precipitation > evapotranspiration) to periodic, high water stress (precipitation < evapotranspiration) [14]. Under this precipitation regime, soil moisture is recharged in pulses resulting in changes in the depth profile for soil moisture and transient resources available for plants [15]. Interspersed between these pulses of precipitation are periods of low water availability with limited resources for plants. As these periods of high water stress become more pronounced, alterations in plant community structure and function can occur. For example, research has demonstrated precipitation-related changes in above- and below-ground plant productivity [16, 17], plant physiology [18, 19], nutrient cycling [20, 21], and community composition [22, 23]. However, the impacts of changing precipitation regimes on plants are typically documented by manipulating the amount of seasonal or annual precipitation, while fewer studies manipulate both the amount and frequency of precipitation to tease apart the relative importance and interactive effects of these factors [9–11]. Variability in precipitation regimes can have different and potentially greater ecological consequences on plants than changes in the amount of precipitation [24, 25].

Experimental manipulations of the amount and/or frequency of precipitation reveal that large, infrequent precipitation events can have important effects on plant performance [16, 26, 27]. For example, Heisler-White et al. [28] added the average seasonal precipitation to three types of plant communities (mesic, semi-arid, and prairie) in 4, 6, and 12 events per month. They found that less frequent but larger precipitation events caused an 18% decrease in above-ground net primary productivity in the mesic grassland, and a 30% and 70% increase in the semi-arid and prairie grassland, respectively. Similarly, Fay et al. [29] examined the response of plant communities to variation in the amount and frequency of precipitation. They found that above-ground net primary productivity and photosynthesis were positively affected, while soil CO_2_ efflux was negatively affected by increased precipitation variability, but which factor (i.e.,frequency vs. amount of precipitation) drove the response varied according to the metric measured. Schneider et al. [30] examined the responses of two mesic grassland species (*Elymus repens* [formerly *Agropyron repens*] and *Lupinus perennis*) to changes in the amount and frequency of precipitation. They found that decreasing the frequency of precipitation reduced biomass in both species, whereas reduced precipitation volume resulted in species-specific responses. The variation in results among these studies illustrates the need for further study of the magnitude and direction of plant responses to projected climate change in order to predict community- and ecosystem-level changes to precipitation variability.

Distinguishing between plant functional groups and studying plant functional traits could provide a predictive explanation for plant responses to precipitation variability. Grasslands are composed of grasses and eudicots, which represent two plant functional groups that differ in morphology, physiology and evolutionary history. Typically, grasses have a small leaf area, vertical leaf orientation and often shallow rooting depths, whereas most eudicots have a larger leaf area, horizontal leaf orientation, and a greater diversity of rooting depths [31, 32]. Also, these groups of species have distinct photosynthetic pathways (e.g., C3 vs C4 photosynthesis) and transpiration rates that enable different water use efficiencies [33, 34]. These characteristics could predict a species’ response to variation in precipitation because certain traits (e.g., shallow rooting depth) make certain plant species vulnerable to environments with periodic, high water stress. Therefore, classifying plant species by their functional group and functional traits could help to predict how the composition of a plant community will change in response to projected precipitation variability.

The objective of this study was to experimentally examine the impacts of variable precipitation on the performance of 14 common native grassland plant species. These species encompassed both graminoid monocots and eudicots and were grown individually under rainout shelters in southern Ontario. This region is forecasted to experience larger, infrequent precipitation events in the next century [1, 35]. After assessing how the amount and frequency of precipitation affected soil moisture variability, we addressed two specific research questions: (i) How does the amount and frequency of precipitation affect plant performance? (ii) Do plant functional types vary in their response to precipitation variability? Addressing these questions will provide empirical evidence about how plant performance will change under different precipitation regimes. This will be important for forecasting changes in plant community composition, and ultimately the maintenance and persistence of grassland biodiversity.

## Materials and Methods

### Study system

We conducted the experiment in an old field meadow at University of Toronto Mississauga in Mississauga, Ontario, Canada (43°32' N, 79°39' W) with their permission to use their property. The field site is dominated by *Dactylis glomerata*, *Plantago lanceolata*, *Phleum pratensis*, *Solidago* spp., *Taraxacum officinale*, and *Trifolium pratense*. The soils consist of a sandy loam topsoil and a sandy loam-clay subsoil horizon. During the experimental period of May to August 2014, total precipitation at the field site was 254 mm (63.5 mm/month) and average temperature was 18.0°C (range = 1.8°C– 32.0°C) (data: University of Toronto Mississauga Meteorological Station; http://www.utm.utoronto.ca/geography/resources/meteorological-station).

At the field site, we planted 15 grassland species (one did not survive) that represent a wide diversity of monocots and eudicots found in grasslands of southern Ontario. We included five monocots: *Andropogon gerardii* Vitman (Poaceae), *Elymus canadensis* L. (Poaceae), *Elymus riparius* Wiegand (Poaceae), *Panicum virgatum* L. (Poaceae), and *Sorghastrum nutans* (L.) Nash (Poaceae), but *S*. *nutans* failed to grow. We included 10 eudicots: *Asclepias tuberosa* L. (Asclepiadaceae), *Desmodium canadense* (L.) DC (Fabaceae), *Eupatorium perfoliatum* L. (Asteraceae), *Euphorbia corollata* L. (Euphorbiaceae), *Geum triflorum* Pursh (Rosaceae), *Oenothera biennis* L. (Onagraceae), *Penstemon hirsutus* (L.) Willd (Scrophulariaceae), *Solidago nemoralis* Aiton (Asteraceae), *Verbena stricta* Vent. (Verbenaceae), and *Zizia aurea* (L.) W.D.J. Koch (Apiaceae). Seeds were obtained from fields in southern Ontario.

### Experimental Design

We employed a fully factorial, randomized, complete block design to examine the effects of changing the amount and frequency of precipitation on plant performance. In May 2014, we established 10 rainout shelters that each covered a 6 m^2^ area, with 1 m spacing between shelters (S1 Fig). The frame of the rainout shelter was constructed of four wooden corner posts anchored 0.6 m into the ground (S2a Fig). Two adjacent posts stood at 1.8 m above the soil surface, while the other two adjacent posts stood at 1.2 m above the soil surface. To construct the rainout shelter’s roof, we secured a wooden square frame with a middle support beam to the four corner posts that allowed the roof to sit on an angle of 45°. Overtop of the rainout shelter’s roof, we attached 6 mil clear polysheeting (Uline, Brampton, Ontario, Canada) that transmitted ca. 90% of photosynthetically active radiation. To the lowest side of the rainout shelter, we connected an eaves trough to collect ambient rainfall into a bucket that added an additional 0.3 m to the shelter. The rainout shelters and eaves troughs covered 2.4 m × 2.7 m, while our plants were planted within a 1.6 m × 1.9 m area which gave them a 0.4 m buffer on all sides that reduced exposure to ambient precipitation [36]. Each rainout shelter contained 6 plots that were 0.38 m × 0.76 m (w × l) with 0.3 m spacing between plots (S2b Fig). We randomly assigned one of the six precipitation regimes (see below) to each plot. To control for lateral water flow between blocks and plots, each plot contained 18–5 L free draining pots (0.127 m × 0.127 m × 0.305 m; w × l × d) that were sunken into the ground at a depth of 0.25 m and arranged in a 3 × 6 rectangular grid. To these 18 pots, we randomly assigned the 15 study species and left three pots without plants for soil volumetric moisture content (VMC) measurements (see below). We did not measure VMC from pots containing plants to avoid damaging plants during the experiment. Nevertheless, our measures of soil moisture should accurately reflect temporal dynamics in water available to plants. In May 2013, seeds obtained from fields in southern Ontario were planted in 0.06 L pots and grown in a greenhouse in St. Williams, Ontario, Canada (42°41'N, 80°26'W). In May 2014, we transferred one year old plants grown in a greenhouse from seed in 0.06 L pots since May 2013 in St. Williams, Ontario, Canada (42°41'N, 80°26'W) to the field site. We removed plants from their original pots, loosened the soil and roots, and transplanted the plants individually into pots using the soil at the field site (a sandy loam) and 0.5 g of slow-release fertilizer (Smartcote, N:P:K, 14:14:14, Brantford, Ontario, Canada).

Our experiment consisted of 10 replicates per precipitation regime of each study species, with one replicate plant of each species in a plot and six replicates of each species in a block (S1 Fig). Our experiment also consisted of 30 replicates per treatment of soil VMC, with three replicates per plot and 18 pots containing no plants (three per plot × six plots) per block. In total, the experiment included 900 plants and 180 pots containing no plants for soil VMC.

### Precipitation regimes

To all plants we imposed a factorial manipulation of the amount and frequency of precipitation (S1 Table). The amount of precipitation was manipulated by giving plants either 70 mm/month or 90 mm/month of water. The frequency of precipitation was manipulated by adding water in equal volumes every 3, 15, or 30 days to reach the monthly volume treatment (70 mm/month or 90 mm/month). To prevent mortality in the 30 day treatment, we added 5 mm every 7 days for the 70 mm/month treatment or 5 days for the 90 mm/month treatment. We applied precipitation treatments between 30 May 2014 and 27 August 2014 by manually adding tap water to the soil surface. Large events were applied over a 2 to 3 h period so that plants never received more than 20 mm of precipitation per hour, which minimized run-off and evaporation from water pooling at the surface.

Our six regimes were based on precipitation records from 1938 to 2012 (data: Government of Canada; http://climate.weather.gc.ca), with 70 mm/month representing the average monthly precipitation amount received in Mississauga, Ontario, Canada, for the last 75 years. In this region, the number of large, infrequent summer precipitation events has and is likely to continue to increase [1, 35]. Our precipitation regimes reflect realistic scenarios that have low recurrence in our historical precipitation data.

### Soil and plant performance measurements

Throughout the experiment, we measured soil VMC to quantify soil moisture variability. Using a soil core that was 6 cm deep and 5 cm in diameter, we removed soil from pots without plants, immediately weighed the sample in the lab, oven dried the sample for 24 h at 100°C, and then weighed the dry sample. We collected a randomly chosen set of three cores on a weekly basis and also at the start of a 30 day cycle for all precipitation regimes, as well as three cores before and after precipitation events with frequencies of 15 and 30 days. Throughout the duration of the experiment, we returned to unplanted pots at most three times to collect soil VMC measurements. We averaged the three core measurements to obtain a weekly measurement.

To assess plant performance, we measured plant morphological and physiological traits. Near the beginning (26 May 2014) and end (22 August 2014) of the experiment we measured leaf number and plant height. We do not show leaf number results because they reveal few changes (S2 Table). On four occasions (2–4 July, 9–11 July, 23–25 July, 31 July—1 August 2014), we used a Delta-T AP4 Porometer (Delta-T Devices, Burwell, Cambridge, UK) to measure stomatal conductance on a fully expanded top leaf for six of the 10 replicates for all treatments and species. Stomatal conductance quantifies the rate of water vapour loss from a leaf through its stomata, and thus provides a measure of a plant’s physiological responsiveness to water stress. On a few occasions a middle or bottom leaf was used because top leaves were too small or exhibited herbivore damage. We harvested above-ground biomass on 29 August 2014 and below-ground biomass from 2–4 September 2014. We dried plant tissue in an oven for 72 h at 60°C.

### Statistical analysis

We tested for the effects of the precipitation regimes on metrics of plant performance using R 3.1.2 [37]. Our predictor variables were amount (70 vs 90 mm), frequency (3, 15 or 30 days), and the interaction amount × frequency, which were all treated as fixed effects in the models. Plot nested within rainout shelter was included as a random effect in the model with a fixed intercept. Our response variables were soil moisture and plant performance including above- and below-ground biomass, above:below-ground biomass ratio, plant height, and stomatal conductance. All analyses were performed using the ‘lmer’ function in the ‘lme4’ package [38]. We tested for the significance of our predictor variables using the ‘anova’ function and type III sums-of-squares in the ‘lmerTest’ package [39], with Kenward-Roger denominator degrees of freedom calculated with the ‘pbkrtest’ package [40]. To improve normality and homogeneity of variance, we log- or square root-transformed our data when necessary. The coefficient of variation was calculated by dividing the standard deviation by the mean.

## Results

### Soil moisture

**Fig 1:**
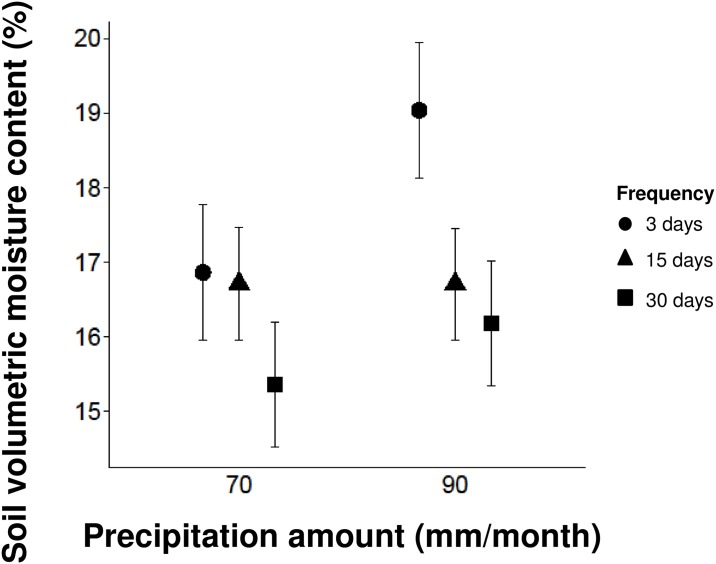
Soil volumetric moisture content in response to precipitation amount and frequency. The effects of precipitation amount and frequency on soil volumetric moisture content. There was a significant effect of frequency on soil moisture (F_2,48_ = 3.45, P = 0.04), with soil moisture decreasing with less frequent precipitation. There was no significant effect of either precipitation amount or the amount × frequency interaction (P>0.10). Points show least-squares mean values and whiskers denote ± 1 SE around the mean.

Manipulating the frequency of precipitation events rather than the amount of monthly precipitation drove changes in soil moisture (Fig 1, S3 Fig). Decreasing precipitation frequency from 3 to 15 days and from 3 to 30 days caused a decrease in soil VMC by 7% and 12%, respectively (F_2,48_ = 3.45, P = 0.04). The amount of precipitation did not affect soil VMC (F_1,48_ = 2.38, P = 0.13), and the amount and frequency of precipitation did not interact (F_2,45_ = 0.96, P = 0.39). As expected, the coefficient of variation in soil VMC was lowest for the 3 day frequency precipitation treatment (70 mm: 18%, 90 mm: 21%), and greatest for the 15 day (70 mm: 37%, 90 mm: 35%) and 30 day (70 mm: 34%, 90 mm: 29%) frequency treatments. The latter two treatments show similar coefficients of variation suggesting that soil moisture variability was likely muted by the small precipitation events that intervened the large 30 day events.

### Plant performance

Manipulating the amount and frequency of precipitation affected multiple components of plant performance, with larger effects on eudicots than monocots (Table 1). Our precipitation regimes affected at least one measure of plant performance in two of the four (50%) monocot species and six of the ten (60%) eudicot species. Of the 14 significant effects detected on morphological traits, four of these were caused by changes in precipitation amount, four were attributed to changes in precipitation frequency, and six resulted from the interaction between the amount and frequency of precipitation. Of the 19 significant effects on physiological traits, two of these were caused by changes in precipitation amount, 15 of these were due to the effect of precipitation frequency, and two of these resulted from an interaction between the amount and frequency of precipitation.

**Table 1:** The effect of the amount and frequency of precipitation on plant performance. The effects of precipitation amount and frequency on plant performance. For each of the fourteen species (4 monocots and 10 eudicots) we consider how plant height, above-ground biomass, below-ground biomass and the ratio of above:below-ground biomass are affected by precipitation amount (70 vs 90 mm), precipitation frequency (3, 15 or 30 days), and the amount × frequency interaction. Linear mixed-effects models were used in all analyses and we report the numerator degrees of freedom (ndf), the denominator degrees of freedom (ddf), F-values, and P-values for all effects. A significant relationship (P < 0.05) is shown in bold.

	**Monocots**																								
	***Elymus riparius***					***Elymus canadensis***					***Andropogon gerardii***					***Panicum virgatum***									
		ndf	ddf	*F*	*P*		ndf	ddf	*F*	*P*		ndf	ddf	*F*	*P*		ndf	ddf	*F*	*P*					
**Plant height**																									
Amount	log	1	44.15	0.796	0.377		1	45	1.611	0.211		1	45	0.885	0.352		1	42.766	0.359	0.552					
Frequency		2	44.147	1.960	0.153		2	45	1.152	0.325		2	45	2.194	0.123		2	42.93	0.334	0.718					
Amount X Frequency		2	44.147	0.542	0.586		2	45	1.244	0.298		2	45	2.436	0.099		2	42.93	0.037	0.964					
**Above-ground biomass**																									
Amount		1	41.45	0.925	0.342	sqrt	1	44.296	0.147	0.703	sqrt	1	45	0.008	0.931		1	41.475	2.276	0.139					
Frequency		2	41.593	0.332	0.719		**2**	**44.29**	**3.776**	**0.031**		2	45	1.695	0.195		2	41.519	0.404	0.670					
Amount X Frequency		2	41.593	1.791	0.180		2	44.29	0.691	0.506		2	45	0.373	0.691		2	41.432	1.431	0.251					
**Below-ground biomass**																									
Amount		1	41.555	1.583	0.215		1	44.296	1.057	0.310		1	45	0.362	0.550	sqrt	1	41.37	1.133	0.293					
Frequency		2	41.723	2.810	0.072		2	44.29	2.088	0.136		2	45	0.347	7.088		2	41.405	0.193	0.826					
Amount X Frequency		2	41.723	2.461	0.098		2	44.49	1.420	0.253		2	45	0.378	0.688		2	41.335	0.119	0.888					
**Above:Below-ground biomass**																									
Amount	sqrt	1	41.779	0.082	0.775	sqrt	1	44.235	0.438	0.512	log	1	45	1.165	0.286	log	1	41.473	0.256	0.615					
Frequency		2	41.987	1.762	0.184		2	44.23	0.234	0.792		2	45	0.444	0.644		2	41.56	0.359	0.700					
Amount X Frequency		**2**	**41.987**	**8.869**	**0.001**		2	44.23	2.867	0.067		2	45	0.137	0.872		2	41.429	1.817	0.175					
	**Eudicots**																								
	***Desmodium canadense***					***Geum triflorum***					***Zizia aurea***					***Penstemon hirsutus***					***Eupatorium perfoliatum***				
		ndf	ddf	*F*	*P*		ndf	ddf	*F*	*P*		ndf	ddf	*F*	*P*		ndf	ddf	*F*	*P*		ndf	ddf	*F*	*P*
**Plant height**																									
Amount	log	1	45	0.506	0.481	sqrt	1	42.7	1.173	0.285	log	1	43.312	3.245	0.079		1	45	0.107	0.745		1	44.226	0.231	0.633
Frequency		2	45	1.107	0.339		**2**	**42.636**	**2.993**	**0.026**		2	43.372	0.146	0.864		2	45	1.836	0.171		**2**	**44.221**	**3.407**	**0.042**
Amount X Frequency		2	45	0.502	0.609		**2**	**42.685**	**3.984**	**0.026**		2	43.372	1.290	0.286		2	45	0.671	0.516		2	44.221	2.891	0.066
**Above-ground biomass**																									
Amount		**1**	**44.145**	**5.360**	**0.025**	log	**1**	**43.291**	**4.252**	**0.045**	log	**1**	**43.515**	**8.784**	**0.005**	log	1	44.296	1.060	0.309		1	43.33	1.185	0.282
Frequency		2	44.142	0.868	0.427		2	43.349	1.638	0.206		2	43.595	0.435	0.650		2	44.29	0.644	0.530		2	43.392	0.911	0.410
Amount X Frequency		2	44.142	1.971	0.151		2	43.349	1.946	0.155		2	43.595	0.357	0.702		2	44.29	0.006	0.994		2	43.392	2.681	0.080
**Below-ground biomass**																									
Amount		1	44.062	1.776	0.190	log	1	43.325	2.498	0.121	log	**1**	**43.441**	**6.650**	**0.013**	sqrt	1	44.219	1.414	0.241	log	1	43.163	3.105	0.085
Frequency		2	44.06	0.097	0.908		2	43.386	0.082	0.922		2	43.515	0.147	0.864		2	44.215	0.061	0.941		2	43.199	0.635	0.535
Amount X Frequency		2	44.06	0.901	0.414		2	43.386	0.172	0.843		2	43.515	0.033	0.967		2	44.215	3.048	0.058		**2**	**43.199**	**4.499**	**0.017**
**Above:Below-ground biomass**																									
Amount	sqrt	1	44.24	3.677	0.062	log	1	43.352	0.000	0.990	log	1	43.515	0.275	0.603	log	1	44.296	2.621	0.113	log	1	43.289	0.818	0.371
Frequency		2	44.235	0.494	0.613		2	43.417	0.683	0.511		2	43.595	0.780	0.465		2	44.29	0.059	0.942		2	43.346	1.035	0.364
Amount X Frequency		2	44.235	1.119	0.336		2	43.417	0.890	0.418		2	43.595	0.276	0.760		2	44.29	2.219	0.121		**2**	**43.346**	**5.353**	**0.008**
	***Verbena stricta***					***Asclepias tuberosa***					***Oenothera biennis***					***Euphorbia corollata***					***Solidago nemoralis***				
		ndf	ddf	*F*	*P*		ndf	ddf	*F*	*P*		ndf	ddf	*F*	*P*		ndf	ddf	*F*	*P*		ndf	ddf	*F*	*P*
**Plant height**																									
Amount		1	41.621	0.328	0.570	log	1	44.121	1.481	0.230		1	45	0.463	0.500	sqrt	1	43.425	0.583	0.449		1	43.607	2.763	0.104
Frequency		2	41.041	0.490	0.616		**2**	**44.118**	**6.833**	**0.003**		2	45	0.045	0.956		2	43.498	1.170	0.320		2	43.571	0.335	0.717
Amount X Frequency		2	41.436	2.311	0.112		**2**	**44.118**	**4.142**	**0.022**		**2**	**45**	**3.681**	**0.033**		2	43.498	0.328	0.722		2	43.517	0.378	0.688
**Above-ground biomass**																									
Amount	sqrt	1	41.639	3.744	0.060	log	1	43.019	0.034	0.854	sqrt	1	44.112	0.200	0.657		1	41.737	1.924	0.173		1	44.19	1.450	0.235
Frequency		2	41.055	0.727	0.489		2	42.72	42.720	0.840		2	44.109	1.010	0.373		2	41.714	0.823	0.446		2	44.186	0.518	0.599
Amount X Frequency		2	41.454	2.617	0.085		2	42.952	0.774	0.467		2	44.109	1.181	0.316		2	41.673	0.057	0.945		2	44.186	0.236	0.791
**Below-ground biomass**																									
Amount	sqrt	1	41.639	1.754	0.193	log	1	42.703	0.385	0.538		1	44.149	0.022	0.883		1	42.186	0.010	0.920	sqrt	1	44.225	0.783	0.381
Frequency		2	41.055	0.060	0.942		2	42.474	1.349	0.271		2	44.146	0.294	0.747		2	42.153	1.197	0.312		2	44.22	1.017	0.370
Amount X Frequency		2	41.454	2.905	0.066		2	42.657	0.957	0.392		2	44.146	1.361	0.267		2	42.096	0.972	0.387		2	44.22	1.389	0.260
**Above:Below-ground biomass**																									
Amount		1	41.087	0.326	0.571	log	1	41.677	0.200	0.657	sqrt	1	4.272	0.004	0.948	log	1	42.186	1.726	0.196	sqrt	1	44.155	0.033	0.857
Frequency		2	40.661	1.819	0.175		2	41.193	1.374	0.265		2	44.266	0.317	0.730		2	42.153	2.905	0.066		2	44.152	0.348	0.708
Amount X Frequency		2	40.935	1.279	0.289		2	41.573	0.617	0.544		2	44.266	0.680	0.512		2	42.096	1.243	0.299		2	44.152	0.840	0.438

**Fig 2:**
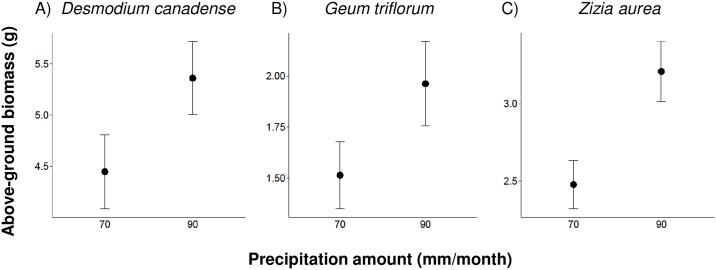
Above-ground biomass in response to precipitation amount and frequency. The effect of precipitation amount on above-ground biomass for three eudicot species. There was a significant effect of precipitation amount on: (A) *Desmodium canadense*, (B) *Geum triflorum*, and (C) *Zizia aurea*, whereas the effects of frequency and amount × frequency interaction were non-significant (see Table 1). Points show least-squares mean values and whiskers denote ± 1 SE around the mean.

**Fig 3:**
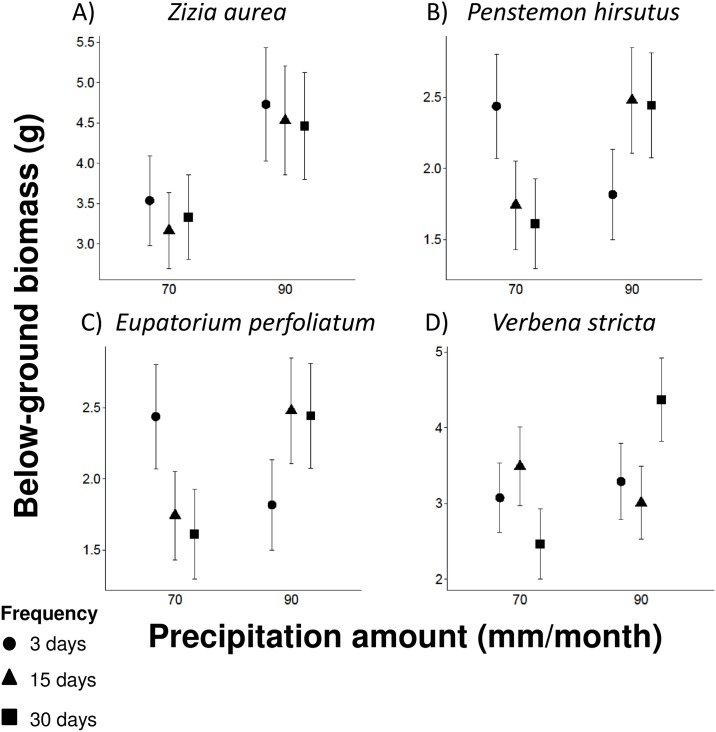
Below-ground biomass in response to precipitation amount and frequency. The effects of precipitation amount and frequency on below-ground biomass. There was a significant effect of precipitation amount on (A) *Zizia aurea*. Precipitation amount and frequency interacted to affect: (B) *Penstemon hirsutus*, (C) *Eupatorium perfoliatum*, and (D) *Verbena stricta* (see Table 1). Points show least-squares mean values and whiskers denote ± 1 SE around the mean.

Plant above- and below-ground biomass was affected by the amount of precipitation or the interaction between the amount and frequency of precipitation. We found increasing the precipitation amount from 70 to 90 mm caused an increase in above-ground biomass in three of the ten eudicot species (Table 1; Fig 2): *Desmodium canadense* (Fig 2a), *G*. *triflorum* (Fig 2b), and *Z*. *aurea* (Fig 2c) increased in biomass by 21%, 30%, and 30%, respectively. We also found that four of the ten eudicot species showed a change in below-ground biomass (Table 1; Fig 3). *Zizia aurea* exhibited a 37% increase in below-ground biomass when precipitation amount was increased from 70 to 90 mm (Fig 3a). In contrast, *P*. *hirsutus*, *E*. *perfoliatum*, and *V*. *stricta* experienced a change in below-ground biomass due to the interaction between the amount and frequency of precipitation. *Penstemon hirsutus* below-ground biomass decreased by 28–34% in the 70 mm treatment and increased by 34–36% in the 90 mm treatment when event frequency decreased from 3 to 15 or 30 days (Fig 3b). *Eupatorium perfoliatum* below-ground biomass decreased by 18% in the 70 mm-15 day regime and increased by 40% in the 90 mm-15 day regime relative to the 3 day treatment (Fig 3c). *Verbena stricta* below-ground biomass decreased by 20% in the 70 mm-30 day regime and increased by 33% in the 90 mm-30 day regime relative to the 3 day treatment (Fig 3d). Thus, increasing the amount of precipitation, even when the frequency of precipitation events is reduced, generally leads to higher plant performance than average precipitation amount and frequency.

Our results show that the relative allocation of above- and below-ground biomass (i.e., above:below-ground ratio) for *E*. *perfoliatum* and *E*. *riparius* was strongly affected by the interaction between the amount and frequency of precipitation (Table 1). With decreasing event frequency, the above:below-ground biomass ratio of *E*. *perfoliatum* increased by 16–19% in the 70 mm treatment (mean ratio: 3 day = 2.07, 15 day = 2.47, 30 day = 2.40) and decreased by 23–37% in the 90 mm (3 day = 2.73, 15 day = 1.73, 30 day = 2.09), relative to the 3 day treatment. The above:below-ground biomass ratio of *E*. *riparius* increased by 33% in the 70 mm-30 day regime relative to the 3 day treatment (3 day = 0.86, 15 day = 0.81, 30 day = 1.14), and decreased by 22–55% in the 90 mm treatment with decreasing event frequency (3 day = 1.33, 15 day = 1.04, 30 day = 0.59). These changes were driven by an increase in above-ground biomass in the 70 mm treatment and decrease in above-ground biomass in the 90 mm treatment with decreasing event frequency.

**Fig 4:**
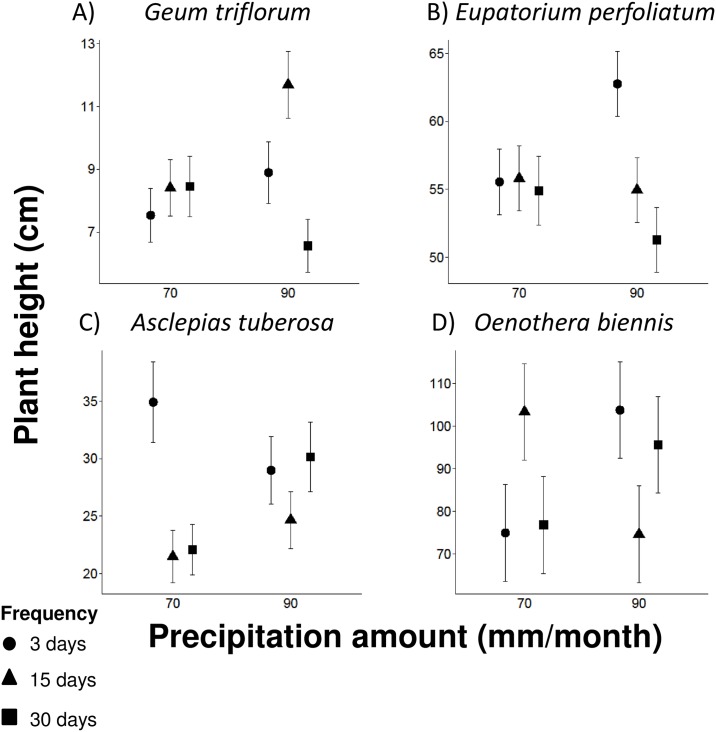
Plant height in response to precipitation amount and frequency. The effects of precipitation amount and frequency on plant height. Precipitation amount and frequency interacted to affect: (A) *Geum triflorum*, (B) *Eupatorium perfoliatum*, (C) *Asclepias tuberosa*, and (D) *Oenothera biennis*. The main effect of precipitation frequency also affected the species depicted in A-C, whereas the main effect of precipitation amount did not significantly affect any species. Points show least-squares mean values and whiskers denote ± 1 SE around the mean.

Plant height was influenced by the main effect of precipitation frequency and the interaction between the amount and frequency of precipitation (Table 1; Fig 4). *Geum triflorum* experienced a 31% increase in height in the 90mm-15 day treatment and 26% decrease in the 90 mm-30 day treatment, relative to the 3 day treatment (Fig 4a); there was no effect of precipitation frequency in the 70 mm treatment. *Eupatorium perfoliatum* was 12–18% taller in the 90 mm-3 day treatment compared to the 15 and 30 day treatment (Fig 4b); there was again no effect of precipitation frequency in the 70 mm treatment. *Asclepias tuberosa* showed a decrease in plant height by 37% as event frequency decreased in the 70 mm treatment but exhibited no change in height across precipitation frequencies in the 90 mm treatment (Fig 4c). Lastly, *O*. *biennis* plant height increased by 38% in the 70 mm-15 day regime and decreased by 28% in the 90 mm-15 day regime relative to the 3 day treatment (Fig 4d).

Precipitation frequency had the largest effect on stomatal conductance, with nine species showing a significant response (seven eudicots and two monocots) (S3 Table). On nine occasions stomatal conductance was observed to decrease with reduced precipitation frequency. On three occasions stomatal conductance increased as precipitation frequency decreased.

## Discussion

Our experimental test of the effects of the amount and frequency of precipitation on 14 monocot and eudicot grassland plant species reveals four key findings. First, increasing the total amount of precipitation resulted in increased plant biomass for multiple species (Table 1, Figs 2 and 3). Second, decreasing the frequency of precipitation often led to decreased plant performance, and frequent changes in plant physiology (Table 1, S3 Table). Third, the interaction between the amount and frequency of precipitation typically caused a decrease in plant performance in the 70 mm treatment and an increase in plant performance in the 90 mm treatment, with decreasing event frequency (Figs 3 and 4). Lastly, our precipitation regimes impacted both monocots and eudicots, with the latter group experiencing larger and more frequent responses to precipitation variability (Table 1). As a whole, our results reveal that understanding the impact of precipitation variability on plant communities requires the integration of both precipitation amount and frequency, as well as the functional groups and traits of species within plant communities.

### Precipitation amount

Increasing the monthly amount of precipitation caused an increase in above-ground biomass in 30% of the eudicot species but no monocot species (Table 1; Fig 2). This observation supports the well-defined relationship between vegetation response to precipitation, which shows that increased precipitation positively affects above-ground biomass production [41–43]. Larger amounts of precipitation could lead to greater water infiltration of the soil and an increase in the duration of high soil moisture, especially at deeper soil depths where evaporation is low [15, 44]. Therefore plants, especially those with deeper roots, have more access to water resources that promote growth. This could explain why some eudicot species, many of which have deep root systems, showed the greatest response to increased precipitation.

### Precipitation frequency

Infrequent precipitation events lead to periodic drought and lower soil moisture and are thus predicted to cause decreased plant performance [14]. Our study confirmed the expectation that decreasing precipitation frequency results in reduced soil moisture (Fig 1), but this did not always translate into lower plant performance (Table 1; Figs 2 and 3). Changes in precipitation frequency had larger effects on stomatal conductance, an important physiological trait, than allocation to plant biomass. These results reflect the fact that physiological traits respond more quickly to changes in water resources than morphological traits [15, 45]. Our results suggest that rapid physiological responses may allow some plant species to effectively tolerate short-term water shortages associated with infrequent precipitation.

Our measurements of stomatal conductance also provide insight into the physiological mechanisms by which plants may tolerate infrequent precipitation. In some species, we observed an increase in stomatal conductance 2 to 8 days following precipitation, but there was no increase in conductance after 11 days (S3 Table). Our interpretation is that a longer duration between precipitation events decreased soil moisture, which led to an increase in stomatal closure as plants attempted to maintain optimal water potential. A potential trade-off to this water stress avoidance strategy could be a decrease in carbon assimilation, which would result in less biomass production as observed in *E*. *perfoliatum*, *D*. *canadense*, *G*. *triflorum*, and *Z*. *aurea*. Similarly, the 30 day precipitation frequency treatment included small intervening precipitation events, suggesting that for some species regular precipitation events as small as 5 mm were sufficient to maintain stomatal conductance relative to the control. Thus, if large, infrequent precipitation events are supplemented with small precipitation events, plant physiological responses can maintain a non-stressed state and conductance similar to small, frequent precipitation events. This conclusion is consistent with observations from arid and semi-arid grasslands [17, 46], suggesting that mesic grasslands (where our experiment was based) also require small, frequent precipitation events to maintain physiological homeostasis. Further exploration of these physiological responses, including a greater range of physiological measures (e.g., photosynthetic rate, water potential, tissue water content), would provide a mechanistic understanding of how precipitation frequency affects grassland communities, including the ways in which plants tolerate abiotic stress.

### Interactions between the amount and frequency of precipitation

Redistribution of precipitation regimes into large-infrequent rainfall events is expected to decrease plant performance [14], but our study reveals that the relationship between the amount and frequency of precipitation is more complex. As precipitation frequency decreased, 40% of the eudicot species studied exhibited lower below-ground biomass within the 70 mm treatment, while these same species typically increased in below-ground biomass in the 90 mm treatment (Table 1; Fig 3). Thus, large precipitation events can compensate for water stress caused by infrequent rainfall, possibly due to pulses of large rainfall infiltrating deeper soil horizons where evaporative losses are low [15, 44]. An increase in root biomass, as exhibited in the 90 mm treatment, facilitates water uptake and further contribute to an increase in plant performance. This result is important because some climate models in the region of the study forecast that precipitation will increase by 10–20% [1], and thus our results show how some plants can maintain their performance under these predicted climate scenarios.

The interaction between precipitation amount and frequency drove disparate responses in plant height among four species (Fig 4). Plant height increased in some species and decreased in other species with decreasing precipitation frequency, and this variation depended on the amount of precipitation. This variability is difficult to explain, but it could be influenced by competition for light from neighbouring individuals late in the growing season when species reached larger sizes. This suggests that changes in individual species grown alone do not necessarily reveal what will happen in a mixed plant community. Therefore, it may be important to consider other biotic and additional abiotic factors (e.g. plant-plant interactions, soil type, temperature, humidity) to fully understand the impacts of precipitation variability. In particular, elucidating how the individualistic responses of species might change when in competition with other species would be an important next step to explore.

Other studies that have manipulated the amount and/or frequency of precipitation have found similar complex effects of variable precipitation. For example, Fry et al. [23] applied one of three precipitation treatments to mesic grasslands—ambient precipitation, drought, and large-infrequent precipitation events. In the drought and large-infrequent treatments they found an increase in ecosystem respiration and no change in either net CO_2_ ecosystem exchange or photosynthetic rate relative to ambient precipitation. Large-infrequent precipitation events also caused a decrease in species richness and cover, showing that species varied in their tolerance to changes in precipitation as observed in our study. Heisler-White et al. [17] added the average seasonal precipitation amount to a semi-arid plant community in 4 (low frequency), 6 (medium frequency), and 12 (high frequency) events per month. They found that above-ground net primary productivity and photosynthetic rate was greatest in the low precipitation frequency regime and lowest in the high frequency regime. In contrast, leaf water potential showed that plants in the high precipitation frequency regime were more stressed immediately preceding a precipitation treatment. These results combined with our observations of interactions between the amount and frequency of precipitation, show that the effects of variable precipitation on plant physiology and performance are complex and require manipulative experiments to understand how changes to precipitation regimes can influence plant communities.

### Plant functional groups and traits

Changes in the amount and frequency of precipitation had larger and more consistent effects on eudicots than monocots. There are two potential explanations for these changes. First, species that exhibited minimal or no changes have deep root systems that experience less temporal variability in soil moisture in comparison to shallow root systems where competition for water with evaporation is high [47]. For example, *Solidago* spp. such as *S*. *nemoralis* has a rooting depth > 1 m [32]; *A*. *gerardii* experiences rapid growth and produces a root system that branches extensively both horizontally and vertically (> 2 m); and *P*. *virgatum* produces a coarse (3–4 mm) and deep (> 2 m) root system [48]. However, changes in root depth is an unlikely explanation in this scenario because plants were constrained to a pot of 30 cm depth. Second, we observed larger changes in C3 (e.g. *E*. *canadensis* and eudicots) than C4 (*A*. *gerardii* and *P*. *virgatum*) species. C4 species are often more tolerant to changes in water resources because they have higher photosynthetic and lower transpiration rates than many C3 species, which often makes them more efficient at using water [49–51]. This conjecture suggests that C4 plants may become more common with less frequent rain and a decrease in species diversity. This speculation is consistent with the observation that C4 plant lineages are most common in arid regions[52]. Our results suggest that an explicit consideration of plant functional groups and traits could provide a mechanistic understanding and predictive framework for the effects of climate-driven changes in precipitation to plant physiology and demography.

### Conclusions

We experimentally tested the impacts of variable precipitation on plant performance of temperate grassland species and show that changing precipitation regimes can have both positive and negative effects on plant performance. Our study teases apart the relative importance and interactive effects of the amount and frequency of precipitation, while providing a mechanistic explanation for changes in individual species. We found that greater precipitation amount frequently resulted in increased plant performance, whereas decreased precipitation frequency typically reduced plant performance. When precipitation amount and frequency interacted, increasing the amount of precipitation by ca. 20% compensated for the negative effects of the dry period between precipitation events. Also, not all species and traits were affected by the precipitation regimes and our physiological data suggest that rapid physiological responses can mitigate reduced soil moisture in some species. However, the study takes place over one growing season. Therefore we do not know whether species will maintain their response year after year or across different ambient site conditions. Nevertheless, our results are particularly important for conserving and restoring plant communities under environmental stress. Taken together, our study shows that it important to consider both the amount and frequency of precipitation as well as the species and trait composition of communities to predict the response of plant communities to forecasted precipitation regimes.

## Supporting Information

S1 Fig Experimental layout at the field site. The experimental layout at the field site showing how the amount (70 vs 90 mm) and frequency (3, 15, or 30 days) of precipitation were manipulated across experimental units. There were 10 rainout shelters with six plots within each rainout shelter and 18 pots within each plot. Precipitation regimes and species/volumetric soil moisture content were randomly assigned to plots and pots, respectively. Dimensions not to scale.
(TIF)

S2 Fig Rainout shelter at the field site. Images depicting experimental design. A) A rainout shelter at the field site, and B) experimental layout underneath the rainout shelter consisting of six plots (one for each combination of precipitation amount (70 vs 90 mm) and frequency (3, 15, or 30 days) with 18 pots per plot (15 pots for plants and three pots without plants strictly for soil volumetric moisture content measurements). Photo credit: T. Didiano.
(TIF)

S3 Fig Temporal changes in soil volumetric moisture content and precipitation regimes. Changes in soil volumetric moisture content and precipitation from 30 June to 30 August 2014. A-C) Soil volumetric moisture content (%) in which precipitation amount (70 and 90 mm) and frequency (3, 15, or 30 days) were manipulated. Mean soil volumetric moisture content (± 1 SE from the mean) is shown on the far right of each graph. D-F) Precipitation regimes depicting how the precipitation amount (70 and 90 mm) was dispensed within each of the precipitation frequency treatments (3, 15, or 30 days).
(TIF)

S1 Table Summary of precipitation regimes. Summary table of the factorial manipulation of precipitation amount (70 vs 90 mm) and frequency (3, 15 or 30 days) used in the experiment. Precipitation amount was manipulated as either 70 mm/month or 90 mm/month. Precipitation frequency was manipulated by providing equal volumes of water every 3 days, 15 days, or 30 days. The 30 day treatment had smaller precipitation events of 5 mm every 7 days for 70 mm/month, or every 5 days for 90 mm/month. Precipitation regimes were implemented between 30 May 2014 and 27 August 2014. The average precipitation conditions between May and August for Mississauga, Ontario, Canada from 1938 to 2012 are provided at the bottom of the table (Government of Canada; http://climate.weather.gc.ca). A visual depiction of these treatments is provided in S3 Fig.
(DOCX)

S2 Table The effect of the amount and frequency of precipitation on leaf number. The effects of precipitation amount and frequency on leaf number. For each of the fourteen species (4 monocots and 10 eudicots) we consider how leaf number is affected by precipitation amount (70 vs 90 mm), precipitation frequency (3, 15 or 30 days), and the amount × frequency interaction. Linear mixed-effects models were used in all analyses and we report the numerator degrees of freedom (ndf), the denominator degrees of freedom (ddf), F-values, and P-values for all effects. A significant relationship (P < 0.05) is shown in bold.
(DOCX)

S3 Table The effect of precipitation amount and frequency on stomatal conductance. Impact of precipitation regimes on stomatal conductance. For each of the fourteen species (4 monocots and 10 eudicots), we consider how stomatal conductance is affected by precipitation amount (70 vs 90 mm), precipitation frequency (3, 15 or 30 days), and the amount × frequency interaction. Linear mixed-effect models were used in all analyses and we report the numerator degrees of freedom (ndf), the denominator degrees of freedom (ddf), F-values, and P-values for all effects. A significant relationship (P < 0.05) is shown in bold.
(DOCX)
