# Supplementary figures and images for: Disentangling the Effects of Precipitation Amount and Frequency on the Performance of 14 Grassland Species
Source: PLoS One. 2016 Sep 13;11(9):e0162310. doi: 10.1371/journal.pone.0162310 (PMC5021276; doi:10.1371/journal.pone.0162310)

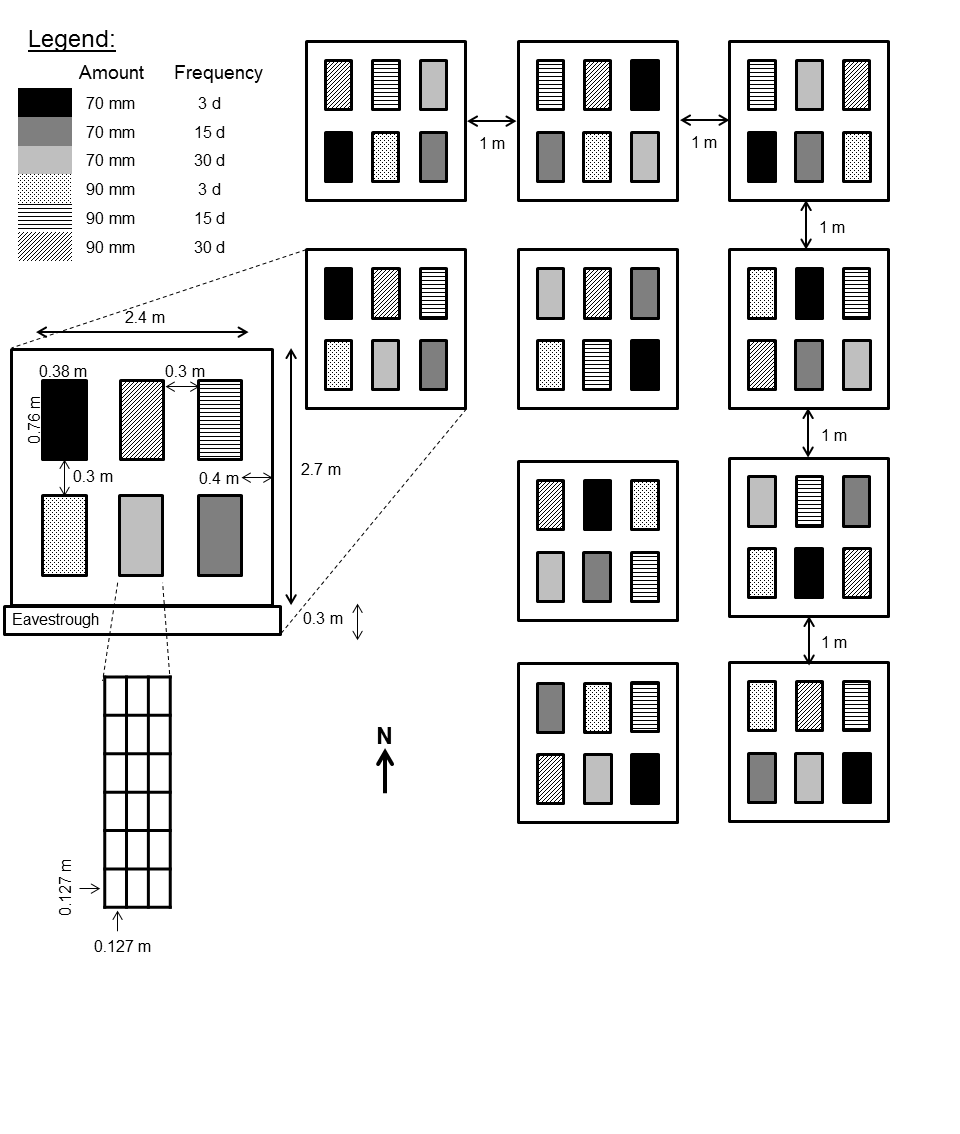

Supplement: S1 Fig — The experimental layout at the field site showing how the amount (70 vs 90 mm) and frequency (3, 15, or 30 days) of precipitation were manipulated across experimental units. There were 10 rainout shelters with six plots within each rainout shelter and 18 pots within each plot. Precipitation regimes and species/volumetric soil moisture content were randomly assigned to plots and pots, respectively. Dimensions not to scale. (TIF) [file pone.0162310.s001.tif]

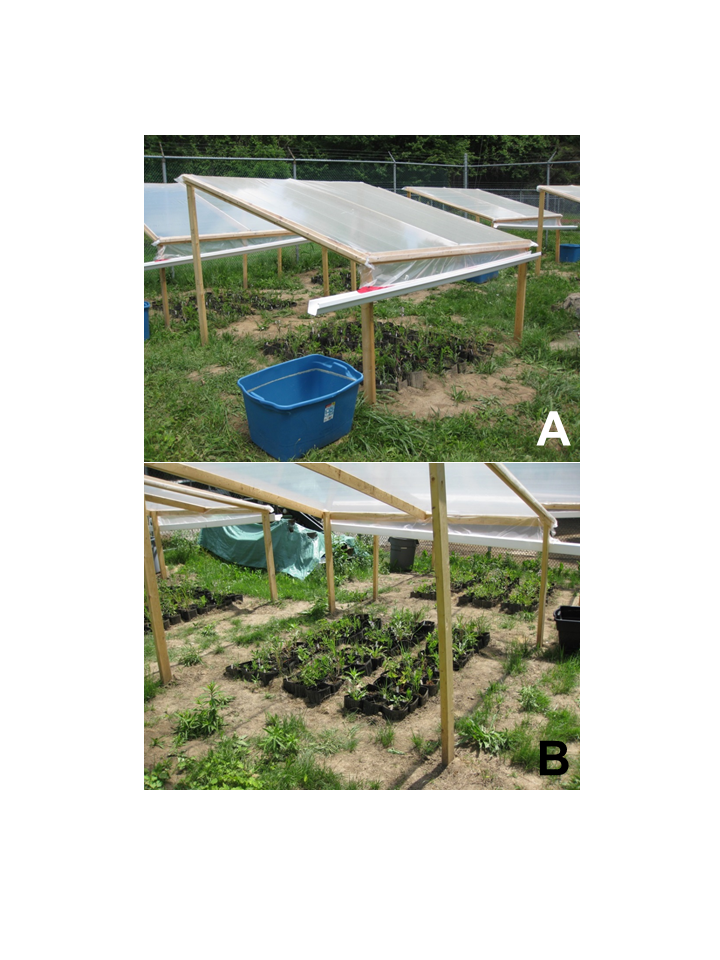

Supplement: S2 Fig — Images depicting experimental design. A) A rainout shelter at the field site, and B) experimental layout underneath the rainout shelter consisting of six plots (one for each combination of precipitation amount (70 vs 90 mm) and frequency (3, 15, or 30 days) with 18 pots per plot (15 pots for plants and three pots without plants strictly for soil volumetric moisture content measurements). Photo credit: T. Didiano. (TIF) [file pone.0162310.s002.tif]

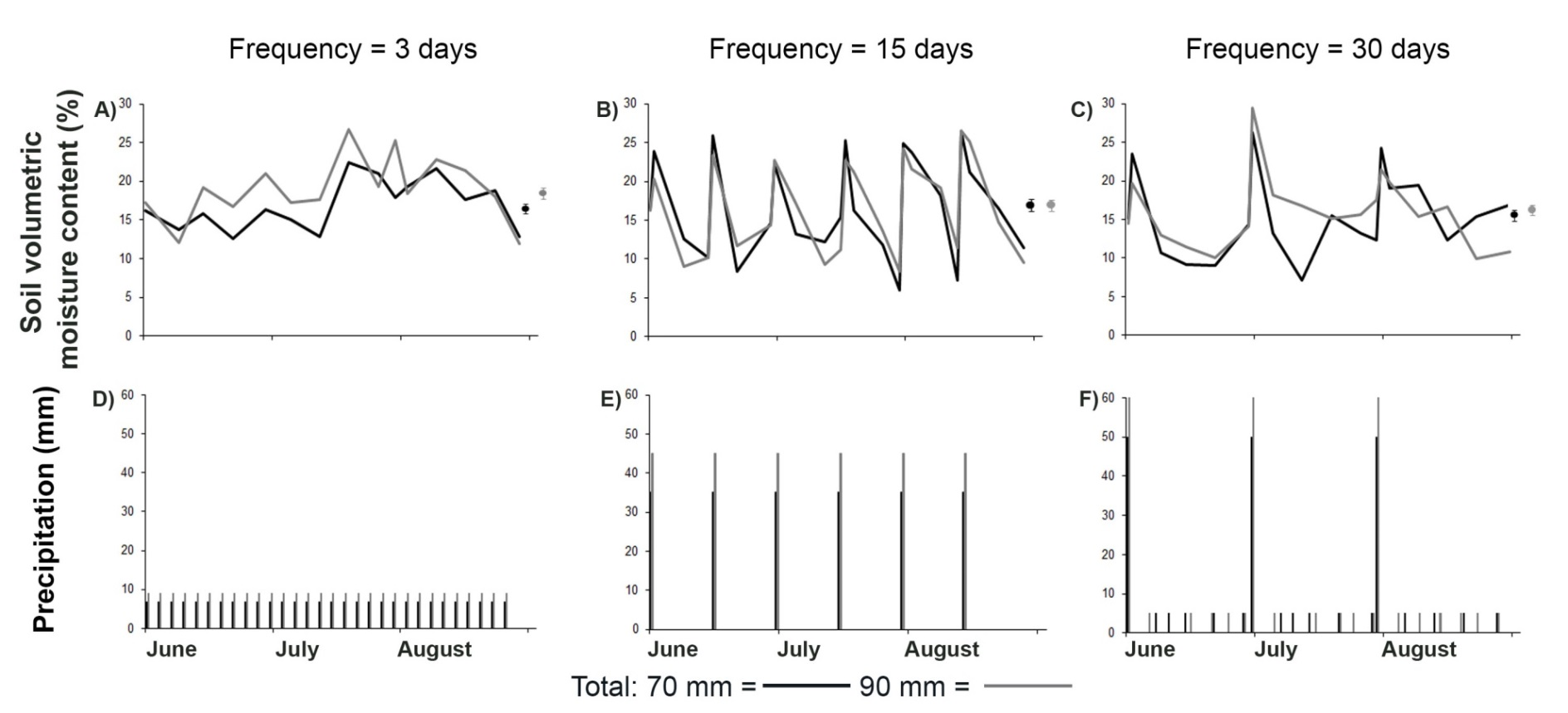

Supplement: S3 Fig — Changes in soil volumetric moisture content and precipitation from 30 June to 30 August 2014. A-C) Soil volumetric moisture content (%) in which precipitation amount (70 and 90 mm) and frequency (3, 15, or 30 days) were manipulated. Mean soil volumetric moisture content (± 1 SE from the mean) is shown on the far right of each graph. D-F) Precipitation regimes depicting how the precipitation amount (70 and 90 mm) was dispensed within each of the precipitation frequency treatments (3, 15, or 30 days). (TIF) [file pone.0162310.s003.tif]
