# Supplementary material for: Disentangling the Effects of Precipitation Amount and Frequency on the Performance of 14 Grassland Species
Source: PLoS One. 2016 Sep 13;11(9):e0162310. doi: 10.1371/journal.pone.0162310 (PMC5021276; doi:10.1371/journal.pone.0162310)
Supplement: S1 Table — Summary table of the factorial manipulation of precipitation amount (70 vs 90 mm) and frequency (3, 15 or 30 days) used in the experiment. Precipitation amount was manipulated as either 70 mm/month or 90 mm/month. Precipitation frequency was manipulated by providing equal volumes of water every 3 days, 15 days, or 30 days. The 30 day treatment had smaller precipitation events of 5 mm every 7 days for 70 mm/month, or every 5 days for 90 mm/month. Precipitation regimes were implemented between 30 May 2014 and 27 August 2014. The average precipitation conditions between May and August for Mississauga, Ontario, Canada from 1938 to 2012 are provided at the bottom of the table (Government of Canada; http://climate.weather.gc.ca). A visual depiction of these treatments is provided in S3 Fig. (DOCX) [file pone.0162310.s004.docx]

| **Precipitation Amount (mm/month)** | **# of Events (per month)** | **Event Amount (mm)** | **Event Frequency (days)** |
| --- | --- | --- | --- |
| 70 | 10 | 7 | 3 |
| 70 | 2 | 35 | 15 |
| 70 | 1 & 4 | 50 & 5 | 30 & 7 |
| 90 | 10 | 9 | 3 |
| 90 | 2 | 45 | 15 |
| 90 | 1 & 6 | 60 & 5 | 30 & 5 |

**Southern Ontario May-August Average Precipitation Conditions 1938-2012**

| 72.57 | 10.27 | 7.07 | 2.99 |
| --- | --- | --- | --- |
