# Supplementary material for: Disentangling the Effects of Precipitation Amount and Frequency on the Performance of 14 Grassland Species
Source: PLoS One. 2016 Sep 13;11(9):e0162310. doi: 10.1371/journal.pone.0162310 (PMC5021276; doi:10.1371/journal.pone.0162310)
Supplement: Supplementary file 3 [file pone.0162310.s005.docx]

Monocots

		A. gerardii					*E. canadensis*					*E. riparius*					*P. virgatum*			
		ndf	ddf	*F*	*P*		ndf	ddf	*F*	*P*		ndf	ddf	*F*	*P*		ndf	ddf	*F*	*P*
**Leaf number**																				
Amount	log	1	45	0.99702	0.3234	log	1	45	0.76036	0.38785		1	43.018	0.0332	0.8563	log	1	42.762	0.50743	0.4801
Frequency		2	45	0.34702	0.7087		2	45	3.13627	0.05307		2	43.159	0.7747	0.4671		2	42.926	1.17904	0.3173
Amount X Frequency		2	45	0.10558	0.9		2	45	0.18255	0.83376		2	43.42	0.15855	0.8539		2	42.926	0.25446	0.7765

Eudicots

		A. tuberosa					*E. perfoliatum*					*P. hirsutus*					*S. nemoralis*					*V. stricta*			
		ndf	ddf	*F*	*P*		ndf	ddf	*F*	*P*		ndf	ddf	*F*	*P*		ndf	ddf	*F*	*P*		ndf	ddf	*F*	*P*
**Leaf number**																									
Amount	log	1	43.596	0.2068	0.65153	log	**1**	**44.296**	**7.8668**	**0.00745**	log	1	45	1.7772	0.1892	sqrt	1	41.244	0.06283	0.8033	sqrt	**1**	**41.639**	**4.3071**	**0.04417**
Frequency		2	43.465	1.3893	0.2601		2	44.29	0.25	0.77991		2	45	2.0357	0.1424		2	41.375	0.12801	0.8802		2	41.055	0.4117	0.66526
Amount X Frequency		**2**	**43.572**	**3.5147**	**0.03845**		2	44.29	0.1723	0.84226		2	45	1.9118	0.1596		2	41.626	0.866125	0.43		2	41.454	2.0537	0.14112

		D. canadense					*E. corollata*					*G. triflorum*					*O. biennis*					*Z. aurea*			
		ndf	ddf	*F*	*P*		ndf	ddf	*F*	*P*		ndf	ddf	*F*	*P*		ndf	ddf	*F*	*P*		ndf	ddf	*F*	*P*
**Leaf number**																									
Amount	sqrt	1	45	2.5098	0.1201		1	43.074	2.20772	0.1446	log	1	43.631	3.9249	0.0539	log	1	45	0.26442	0.6096	log	1	43.323	1.0743	0.3057
Frequency		2	45	1.0267	0.3664		2	43.092	0.22588	0.7988		2	43.595	1.5174	0.2306		2	45	0.05406	0.9474		2	43.385	1.11334	0.3377
Amount X Frequency		2	45	1.8895	0.1629		2	43.092	0.64311	0.5306		2	43.54	2.2319	0.1195		2	45	1.23669	0.3		2	43.385	0.08997	0.9141
