# Supplementary material for: Disentangling the Effects of Precipitation Amount and Frequency on the Performance of 14 Grassland Species
Source: PLoS One. 2016 Sep 13;11(9):e0162310. doi: 10.1371/journal.pone.0162310 (PMC5021276; doi:10.1371/journal.pone.0162310)
Supplement: Supplementary file 4 [file pone.0162310.s006.docx]

Monocots

		Elymus riparius					*Elymus canadensis*					*Andropogon gerardii*					*Panicum virgatum*			
		ndf	ddf	*F*	*P*		ndf	ddf	*F*	*P*		ndf	ddf	*F*	*P*		ndf	ddf	*F*	*P*
**2 & 4 July 2014**																				
Amount	sqrt	**1**	**24.288**	**4.797**	**0.038**		1	25	0.010	0.921		1	25	0.000	0.987	log	1	24.288	0.106	0.748
Frequency		2	24.278	0.189	0.829		**2**	**25**	**5.465**	**0.011**		2	25	0.808	0.457		2	24.278	0.436	0.652
Amount X Frequency		2	24.278	2.613	0.094		2	25	0.788	0.466		2	25	1.820	0.183		2	24.278	1.539	0.235
**9 & 11 July 2014**																				
Amount	sqrt	1	25	0.061	0.808	sqrt	1	25	0.002	0.969		1	25	0.029	0.867	sqrt	1	23.445	3.785	0.064
Frequency		2	25	2.295	0.122		2	25	2.829	0.078		**2**	**25**	**4.045**	**0.030**		2	23.581	0.292	0.750
Amount X Frequency		2	25	1.051	0.365		2	25	0.340	0.715		**2**	**25**	**4.548**	**0.021**		2	23.581	2.958	0.071
**25 & 31 July 2014**																				
Amount	log	1	24.288	0.998	0.328		1	25	3.288	0.082		1	25	0.890	0.354	log	1	25	1.598	0.218
Frequency		2	24.278	3.139	0.061		2	25	0.776	0.471		2	25	1.579	0.226		2	25	1.466	0.250
Amount X Frequency		2	24.278	2.299	0.122		2	25	0.026	0.974		2	25	2.509	0.102		2	25	0.451	0.642
**31 July & 1 August 2014**																				
Amount	log	1	25	0.037	0.849	sqrt	1	25	0.385	0.541	log	1	25	0.048	0.828		1	25	0.014	0.908
Frequency		2	25	0.764	0.477		2	25	0.147	0.864		2	25	0.673	0.519		2	25	0.204	0.817
Amount X Frequency		2	25	1.902	0.321		**2**	**25**	**5.408**	**0.011**		2	25	0.270	0.766		2	25	0.313	0.734

Eudicots

		Desmodium canadense					*Geum triflorum*					*Zizia aurea*					*Penstemon hirsutus*					*Eupatorium perfoliatum*			
		ndf	ddf	*F*	*P*		ndf	ddf	*F*	*P*		ndf	ddf	*F*	*P*		ndf	ddf	*F*	*P*		ndf	ddf	*F*	*P*
**2 & 4 July 2014**																									
Amount	log	1	23.279	3.135	0.090	sqrt	1	22.198	4.515	0.045	sqrt	1	22.621	1.675	0.209		1	24.224	0.600	0.446		1	25	1.050	0.315
Frequency		**2**	**23.384**	**5.801**	**0.009**		2	22.333	0.592	0.561		2	20.627	1.996	0.161		2	24.216	0.567	0.574		2	25	2.187	0.133
Amount X Frequency		2	23.384	0.889	0.424		2	2.333	1.164	0.331		2	20.529	1.477	0.252		2	24.216	0.694	0.509		2	25	1.332	0.282
**9 & 11 July 2014**																									
Amount	log	1	23.197	0.031	0.862	sqrt	1	23.178	0.179	0.677		1	24.365	0.615	0.441	sqrt	1	24.288	0.147	0.705	log	1	25	2.699	0.113
Frequency		**2**	**23.18**	**7.018**	**0.004**		**2**	**23.163**	**8.867**	**0.001**		**2**	**24.49**	**4.461**	**0.022**		**2**	**24.278**	**9.420**	**0.001**		**2**	**25**	**6.878**	**0.004**
Amount X Frequency		2	23.18	1.791	0.181		2	23.163	2.608	0.952		2	24.9	2.319	0.120		2	24.278	3.198	0.051		2	25	0.099	0.906
**25 & 31 July 2014**																									
Amount	log	1	25	1.438	0.242	sqrt	1	25	0.497	0.487	sqrt	1	24.365	1.124	0.300	log	1	25	2.995	0.096		1	23.293	0.521	0.478
Frequency		**2**	**25**	**9.589**	**0.001**		**2**	**25**	**7.566**	**0.003**		2	24.49	2.362	0.115		2	25	1.463	0.251		**2**	**23.401**	**5.734**	**0.009**
Amount X Frequency		2	25	1.215	0.314		2	25	0.658	0.527		2	24.49	3.043	0.066		2	25	0.467	0.632		2	23.401	1.479	0.248
**31 July & 1 August 2014**																									
Amount	sqrt	1	25	1.809	0.191	sqrt	1	24.134	1.029	0.321		1	24.545	0.245	0.625	sqrt	1	24.173	2.821	0.106	sqrt	1	25	2.932	0.099
Frequency		2	25	2.443	0.107		2	24.129	0.421	0.661		2	24.332	2.536	0.100		2	24.167	0.659	0.527		**2**	**25**	**3.714**	**0.039**
Amount X Frequency		2	25	1.537	0.235		2	24.129	0.405	0.671		2	24.498	0.864	0.434		2	24.167	0.821	0.452		2	25	0.315	0.732

		Verbena stricta					*Asclepias tuberosa*					*Oenothera biennis*					*Euphorbia corollata*					*Solidago nemoralis*			
		ndf	ddf	*F*	*P*		ndf	ddf	*F*	*P*		ndf	ddf	*F*	*P*		ndf	ddf	*F*	*P*		ndf	ddf	*F*	*P*
**2 & 4 July 2014**																									
Amount	sqrt	1	20.127	1.556	0.227							1	25	0.057	0.813		**1**	**22.449**	**4.902**	**0.037**		1	24.108	0.134	0.717
Frequency		2	20.02	0.818	0.455							2	25	2.043	0.151		2	22.384	0.690	0.512		2	24.108	2.727	0.086
Amount X Frequency		2	19.578	1.806	0.191							2	25	0.755	0.408		2	22.384	0.019	0.981		2	24.108	0.610	0.551
**9 & 11 July 2014**																									
Amount		1	24.545	0.048	0.828	sqrt	1	24.545	0.558	0.462		1	25	0.001	0.971	sqrt	1	24.288	0.038	0.847		1	25	2.621	0.118
Frequency		2	24.332	0.901	0.419		2	24.49	0.334	0.720		2	25	2.356	0.116		2	24.278	0.549	0.585		**2**	**25**	**9.033**	**0.001**
Amount X Frequency		2	24.498	0.332	0.720		2	24.407	0.195	0.824		2	25	1.975	0.160		2	24.278	0.096	0.145		2	25	2.932	0.072
**25 & 31 July 2014**																									
Amount		1	22.43	2.057	0.165	log	1	25.151	2.518	0.125		1	25	0.997	0.328		1	24.141	0.287	0.597	sqrt	1	25	2.470	0.129
Frequency		2	22.615	0.537	0.592		2	25.151	2.208	0.131		2	25	0.050	0.951		**2**	**24.136**	**4.257**	**0.026**		**2**	**25**	**6.099**	**0.007**
Amount X Frequency		2	22.924	2.784	0.083		2	25.142	1.368	0.273		2	25	2.628	0.092		2	24.136	1.667	0.210		2	25	0.234	0.793
**31 July & 1 August 2014**																									
Amount	sqrt	1	22.265	0.078	0.783		1	25.194	0.006	0.939		1	25	1.707	0.203		1	24.272	0.479	0.495	log	1	25	0.910	0.349
Frequency		2	22.427	0.195	0.824		2	25.186	1.847	0.178		2	25	0.231	0.796		2	24.262	0.983	0.388		2	25	0.733	0.490
Amount X Frequency		2	22.702	2.836	0.080		2	25.186	0.033	0.968		2	25	1.744	0.195		2	24.262	0.031	0.970		2	25	0.029	0.971
